# Insertion torque is not a good predictor of pedicle screw loosening after spinal instrumentation: a prospective study in 8 patients

**DOI:** 10.1186/1754-9493-4-14

**Published:** 2010-09-03

**Authors:** Bengt Sandén, Claes Olerud, Sune Larsson, Yohan Robinson

**Affiliations:** 1 Uppsala University Hospital, Institute for Surgical Sciences, Department of Orthopedics, SE-751 85 Uppsala, Sweden

## Abstract

**Background:**

Pedicle screw loosening is a major safety concern in instrumented spinal surgery due to loosening with potential pseudarthrosis and possible loss of correction requiring revision surgery. Several cadaver studies have compared insertion torque of pedicle screws with resistance to pullout or cyclic loading. In most of these studies, a correlation has been found between these variables. Clinical studies have been made, comparing insertion torque to bone mineral density or radiological signs of screw loosening. There are no clinical studies comparing insertion torque to extraction torque or other biomechanical parameters in vivo. This study was designed to investigate whether the insertion torque of pedicle screws can be used to predict the purchase of the screws.

**Methods:**

The insertion torque of stainless steel pedicle screws was recorded in eight patients undergoing lumbar fusion surgery with four-screw constructs. Torque gauge manometers were used for the recordings. The implants were removed after one year, and the extraction torque of the screws was recorded.

**Results:**

The mean insertion torque was 76 ± 41 Ncm and the mean extraction torque 29 ± 36 Ncm. The r value was 0.591, suggesting that there was a correlation between the insertion and extraction torque. However, the scattergram revealed that the screws could be divided into two groups, six screws with a high correlation between insertion and extraction torque, and 26 screws where no correlation could be demonstrated.

**Conclusions:**

In this unique human in-vivo study, the insertion torque could not be used to predict the purchase of lumbar pedicle screws one year after implantation. It could be demonstrated that in vivo insertion torque alone is of minor value to estimate pullout strength, and should be combined with or replaced by more accurate measures.

## Introduction

Pedicle screw fixation has become one of the standard methods of spinal instrumentation. The purpose of pedicle screw fixation is to increase the stability of the system in order to achieve spinal fusion or fracture healing. Loosening of the pedicle screws is a common complication, which can lead to pseudarthrosis, and possible reoperation, thus presenting a major patient safety concern. The frequency of screw loosening has varied widely in different studies. In a literature review by Esses et al, the rate of loosening varied between 0.6 and 11% [1]. Other studies have reported rates of loosening between 18% and 27% [2-4]. In order to decrease the frequency of screw loosening, several different concepts have been tried, including altering screw shape and thread design, surface modifications of the screws, and PMMA augmentation of the screw holes [5-10]. It follows that spine surgeons need objective methods to predict screw looseing in order to help them decide when to consider these methods.

Intraoperative recording of the insertion torque of the pedicle screws has been proposed as as an evaluation of the screw purchase [11]. Several experimental studies have demonstrated a positive correlation between the insertion torque of pedicle screws and the purchase of the screws in biomechanical tests [11-14]. Based on these findings, some authors have measured the insertion torque of pedicle screws in the operating room and abandoned the use of pedicle screws if too low insertion torque was recorded [11]. Other authors have not confirmed the correlation between insertion torque and the anchorage of the screws in the clinical situation [15-17]. However, in these studies, the judgement of screw fixation was based on the radiological assessment, and screw loosening was defined as presence of a radiolucent zone around the screw. The presence of a radiolucent zone surrounding a pedicle screw is a predictor of screw loosening, but a loose screw is not always surrounded by a radiolucent zone [18]. Therefore, the frequency of screw loosening may have been underestimated in these studies. In the study by Okuyama et al, it was stated that due to the small number of loosening, it should not be concluded from that study that the insertion torque could not predict loosening of the pedicle screws [15].

To the best of our knowledge, there are no clinical studies where the insertion torque of pedicle screws has been correlated to extraction torque or other biomechanical parameters in vivo. The aim of the present study was to investigate the correlation between the insertion torque of pedicle screws and the extraction torque, recorded one year after the application of the screws.

## Materials and methods

Between November 1997 and June 1999, eight (six women and two men) consecutive patients who were to undergo instrumented one- or two-level lumbar or lumbosacral fusion for degenerative disorders agreed to participate in this study. This study was a methodological pilot investigation to a controlled study, using hydroxyapatite-coated pedicle screws, published elsewhere [19]. After thorough revision of the data quality this previously unpublished but unique dataset was recovered and found worth publishing.

The indications for surgery were spinal stenosis in four patients and spondylolisthesis in the other four. The mean age was 57 ± 12 years, range 40 - 74. Partial or total laminectomy was performed in all patients. There were five two-level fusions and three one-level fusions. Four screws were used in each operation. The vertebrae involved were L3 (6 screws), L4 (6 screws), L5 (12 screws) and S1 (8 screws). The Posterior Fixator System (wrought stainless steel, SAF 2507, Anatomica, Gothenburg, Sweden) was used. The diameter of the screws used was 6 mm, and the length 55-70 mm.

Before surgery and at 3 and 6 month postoperatively all patients received plain radiographs in two planes and two lateral views with cranial and kaudal angulations to document screw placement and loosening. The radiographs were evaluated by a radiologist (MP-M) without knowledge of the insertion torque.

During the study period, the frequency of implant removal was high at our center and all implants were extracted after one year to minimize mechanical irritation by screws. Successful fusion was controlled manually during surgery, and in doubtful cases even by exploration of the fusion masses.

The regional medical ethical committee approved the study.

### Insertion of pedicle screws

Standard anatomical landmarks were used for identification of the pedicles, and fluoroscopy to confirm the position of the screws. A pedicle probe was used for the preparation, and the holes were tapped with a tap of the same diameter as the screw and to the entire depth of insertion. All surgical procedures and all recordings of insertion torque were performed by the same surgeon. The insertion torque was recorded using torque gauge manometers with a range of 5-600 Ncm (Eduard Wille GmbH & Co, Wuppertal, Germany). The recordings of insertion torque were measured as the final torque when the entire threaded portion of the screw had been implanted into bone, making sure that the part of the screw embedded into bone was equally long when the recordings were made. The torque gauge manometers were validated for accuracy with a servo-hydraulic testing machine (Mini Bionix 858, MTS Corp, Minneapolis, MN)

### Extraction of pedicle screws

After a mean of 12.2 months (12 to 13) the instruments were removed. The extraction torque was recorded with the same torque gauge manometers as the insertion torque. The maximum extraction torque was recorded. All extraction procedures and recordings of extraction torque were carried out by the same surgeon.

### Statistical analysis

For the statistical evaluation, two-tailed t-test and simple regression were used. P values of less than 0.05 were considered to be statistically significant. The values given are the mean values ± one standard deviation.

## Results

**Figure 1:**
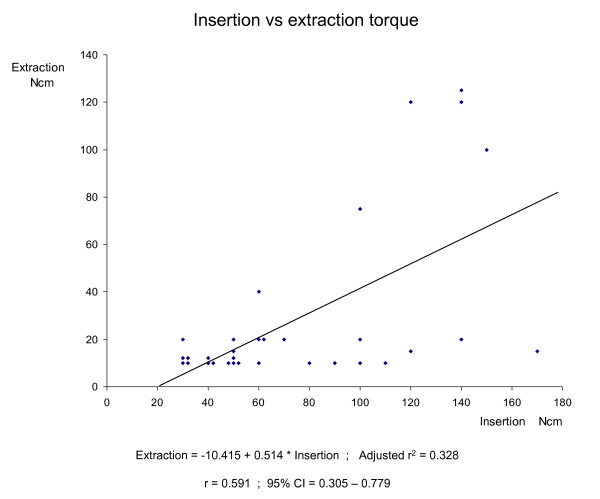
**Insertion vs extraction torque**.

The radiological evaluation following the insertion of the pedicle screws confirmed correct intrapedicular screw placement. No hardware failures such as screw or rod fractures, angulations or disconnections could be noted. The mean insertion torque for the 32 screws was 76 ± 41 Ncm and the mean extraction torque was 29 ± 36 Ncm (p < 0.0001). The insertion torque and the extraction torque were equal for one screw, while the extraction torque was lower than the insertion torque for the remaining 31 screws. Of the 32 screws, only six screws had an extraction torque that exceeded 20 Ncm. These six screws had been implanted in two female patients, 51 years (three screws) and 49 years (three screws), respectively. The calculated r^2 ^value was 0.328 and the r value 0.591 (p = 0.0004), suggesting that there was a correlation between the insertion and extraction torque (Figure 1). However, the scattergram (Figure 1) demonstrates that the screws could be divided into two different groups. Six screws demonstrate a high correlation between insertion and extraction torque, while the remaining 26 screws all have a very low extraction torque and form a line that is parallel to the x-axis. For these 26 screws, there is no correlation between insertion and extraction torque. Theses results remain unchanged if the screws with insufficient insertion torque (40 Ncm or less) are excluded.

## Discussion

The frequency of loosening of pedicle screws probably has been underestimated in many studies [18]. In spite of this, many methods have been developed aiming to reduce the frequency of loosening, and the insertion torque of the screws has been recorded in order to predict loosening [11]. This is based on the assumption that there is a reliable correlation between insertion torque and the long-term anchorage of pedicle screws.

The correlation between insertion torque and the purchase of the screws, recorded as resistance to cyclic loading or pull-out resistance, has been investigated in several ex-vivo studies. In a study on human cadaveric lumbar spines, Zdeblick et al found a linear correlation between insertional torque and number of cycles to failure, while there was no correlation between bone mineral density (BMD) and number of cycles to failure [11]. In another cadaveric study, correlations were found between BMD and insertion torque, BMD and pull-out resistance, and insertion torque and pull-out resistance, respectively. The authors concluded that the maximum insertion torque could predict the mechanical stability [12]. Myers et al found that insertion torque accounted for approximately 60% of the variance in pull-out strength in human cadaveric lumbar vertebrae, and that BMD determined by quantitative computed tomography also was a predictor of pedicle screw purchase, while DXA (dual energy x-ray absorptiometry) was less useful [20]. In a study of sacral screw fixation, using human cadaveric sacrums, insertion torque of pedicle screws was correlated with pull-out strength and stiffness, and it was concluded that insertion torque is a good intraoperative indicator of sacral screw-fixation strength [14].

On the other hand, there are some studies where the correlation between insertion torque and pull-out resistance was less obvious. In a study on human cadaveric lumbar spines and several types of screws, statistically significant correlations could be demonstrated only in some subgroups. The authors concluded that insertion torque is not a good predictor of pull-out strength in cadaveric bone [7]. In a study on calf vertebrae, there was no significant correlation between insertion torque and pull-out strength [21].

There are only a few studies where the insertion torque has been recorded in-vivo. In a study by Bühler et al, the insertion torques were studied for different screw types and correlated to BMD both ex-vivo and in-vivo. The insertion torques were significantly higher in-vivo when compared to cadavers. Several different possible reasons for the difference in insertion torque were discussed, and lubrication from postmortem release of lipids from bone marrow cells was believed to be the most plausible explanation. A significant correlation between insertion torque and BMD could be demonstrated ex-vivo, but not in-vivo [22]. Similar results were found in a study by Mizuno et al, where the correlation between insertion torque and BMD depended on the screw shape, and no correlation was demonstrated for cylindrical screws [17]. However, other in-vivo studies have demonstrated high correlation between insertion torque and BMD [15], and a negative relation between insertion torque and the degree of osteoporosis [16].

In three of these studies, postoperative radiographs were examined and signs of loosening or instability were noted and correlated to the insertion torque [15-17]. However, no correlation could be found between insertion torque and signs of loosening of the screws or insertion torque and signs of instability. In two of the studies it was concluded that intraoperative insertion torque could not be used as an objective predictor of screw loosening or clinical results [16,17]. In the third study, the authors concluded that " it should not be concluded that the insertional torque of pedicle screws cannot intraoperatively predict development of screw loosening" [2]. This was based on the fact that the number of radiographically detected screw loosening was very small, and the authors therefore may have considered that there could have been differences in the purchase of the screws that were not detected with the radiological examinations. It is not very surprising that no correlation could be found between insertion torque and radiological signs of screw loosening, as it has been demonstrated that the frequency of screw loosening is underestimated in radiographs, even if a structured protocol is used for the radiological examinations [18].

In the present study, the insertion torque was correlated to a recording of the purchase of the screws, the extraction torque. The statistical analysis revealed a correlation between insertion and extraction torque with the r value 0.591, but it is obvious that the insertion torque could not predict the purchase of the screws, as most of the screws with good insertion torque were completely loose at removal with an extraction torque of 20 Ncm or less.

The insertion torques recorded in this study are lower than the torques in the other studies of insertion torque in-vivo [15,17,22]. Several different factors could explain these differences. One important reason is the method of preparation of the pedicles. In our study, the holes for the screws were tapped to the entire depth of insertion. Furthermore the screws were tapped line-to-line and not undertapped which can provide higher insertion torques. Another factor influencing the insertion torque is the method of recording, that is how much of the screw that is implanted when the recording is made. Finally, the surface roughness of the screw is important for the torque. The surface roughness of stainless steel is lower than the roughness of titanium, and in one study, the insertion torque of stainless steel and titanium screws were compared. The steel screws had a significantly lower insertion torque than the titanium screws [22]. If different methods for preparation and recording have been used, the values of insertion torque from different studies could not be used for comparisons, i. e. for comparing different screw designs.

The recordings of extraction torque in this study were made one year after implantation of the screws. The ideal situation for evaluating the anchorage of pedicle screws would be mechanical testing at an earlier time-point when the stability of the system is essential, and bone remodeling from soft callus with greater biomechanical instability could be expected. For obvious reasons, recording of extraction torque after such a short time could not be performed on spinal instrumentations used clinically. The resistance to pull-out or cyclic loading probably are better methods than extraction torque for the evaluation of purchase of pedicle screws, but these methods are hardly possible to use clinically. The extraction torque is the best variable that can be used retrospectively for the evaluation of pedicle screw anchorage

With regard to patient safety it would be desirable to predict screw anchorage at the time point of insertion. This would enable the surgeon to decide whether additional means, i.e. screw augmentation, are necessary to achieve the required stability. Unfortunately until now we have no reliable in vivo models for prediction of screw pull-out at different time points. Insertion torques have a weak correlation to extraction torques, as we could show in this study. Furthermore cadaver studies lead obviously to different conclusions with regard to pedicle screw anchorage than in vivo investigations. Therefore we recommend further investigation of newer screw designs, coated screws, and newer alloys in an in vivo setting.

## Competing interests

The authors declare that they have no competing interests.

## Authors' contributions

BS performed the surgeries. BS, CO, SL and YR evaluated the data statistically BS, CO and SL wrote the manuscript, and YR critically revised the manuscript. All authors read and approved the final manuscript.
